# DIET AND NUTRITION: Phosphate Linked to Lung Cancer in Mice

**Published:** 2009-03

**Authors:** Carol Potera

Inorganic phosphate (Pi) is a vital component of membrane phospholipids and nucleotides, both of which provide energy and serve as components of DNA, RNA, and phosphorylated intermediates in cellular signaling. Most living organisms need some Pi to survive, but a diet high in Pi may contribute to lung cancer, according to an animal study reported in the January 2009 issue of the *American Journal of Respiratory and Critical Care Medicine*. The new study by Myung-Haing Cho and colleagues at Seoul National University finds that dietary Pi levels equivalent to those typically found in the modern Western diet were linked with increased lung tumor growth and progression in mice. “The study is the first to demonstrate that dietary phosphates alter the course of cancer in a relevant animal model,” says coauthor George Beck, an assistant professor of endocrinology at Emory University.

Cho and colleagues cite surveys showing that the amount of Pi added to processed foods and beverages increased by about 17% between 1983 and 1993 and may have continued to increase since that time. Pi occurs naturally in foods including cow’s milk, soy products, corn, wheat, eggs, legumes, and chocolate, and food manufacturers also add Pi to many products—including soft drinks, baked goods, cheese products, ice cream, candy, ketchup, mayonnaise, hot dogs, processed meats, and frozen pizzas—to improve water retention and texture.

The researchers selected K-*ras*^LA1^ mice to study the effects of dietary Pi on lung tumors. K-*ras* is the most frequently mutated gene in human tumors, and K-*ras*^LA1^ mutations result in aggressive tumors that resemble non–small cell lung cancer in humans. According to the American Cancer Society’s *Cancer Facts & Figures 2008*, non–small cell lung cancer accounts for 87% of all lung cancer cases. One-month-old mice were fed a lower-Pi diet containing 0.5% phosphorus or a higher-Pi diet containing 1.0% phosphorus (Beck says the latter reflects a moderately elevated yet normal human intake). After 1 month, mice on the higher-Pi diet had twice as many tumors overall as those on the lower-Pi diet and moreover showed a near 3-fold increase in tumors larger than 1.5 mm in diameter (an arbitrary measure chosen to indicate ease of detection).

Changes were also seen at the molecular level. Higher Pi intake was associated with a 3-fold increase in the activity of Akt kinase (a cell-signaling protein that aids tumor growth and makes cancer cells resistant to anticancer therapies) and a doubling in the amount of NPT-2b (a phosphate transport protein). The higher-Pi diet also lowered levels of tumor suppressors including PTEN.

Nevertheless, Beck believes it’s too soon to sound the alarm about Pi-rich foods. “We need to show similar changes in humans before telling people to limit dietary Pi,” he cautions. Such studies should track long-term dietary habits to see if low- and high-Pi intakes relate to lung cancer incidence in human populations. “It would also be interesting to test whether low-Pi diets make existing cancer drugs more effective,” Beck adds.

John Heffner, a pulmonary and critical care physician and director of medical education at Providence Portland Medical Center, says the findings could shed light on questions such as why some smokers develop lung cancer later than others or not at all. Heffner agrees that it’s premature to cut back on Pi-containing food additives or to limit intake of foods that are naturally high in the mineral. “Before warning the public,” he says, “the Food and Drug Administration should review the increase in dietary Pi in light of studies like this.”

## Figures and Tables

**Figure:**
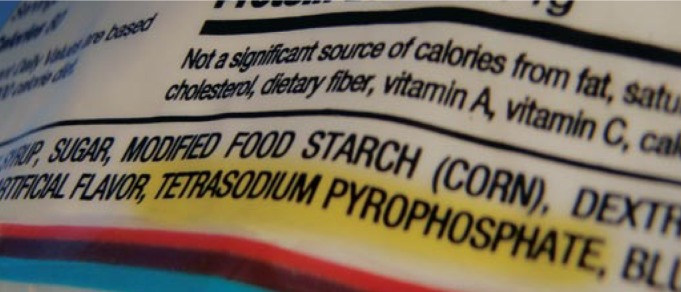
Dietary Pi findings in mice cannot yet be applied to human intake.

